# Assessment Effect of Nanometer-Sized Al_2_O_3_ Fillers in Polylactide on Fracture Probability of Filament and 3D Printed Samples by FDM

**DOI:** 10.3390/ma16041671

**Published:** 2023-02-16

**Authors:** Anton Smirnov, Pavel Peretyagin, Nikita Nikitin

**Affiliations:** 1 Laboratory of 3D Structural and Functional Engineering, Moscow State University of Technology “STANKIN”, Vadkovsky per. 1, Moscow 127055, Russia; 2 Spark Plasma Sintering Research Laboratory, Moscow State University of Technology “STANKIN”, Vadkovsky per. 1, Moscow 127055, Russia; 3 Scientific Department, A.I. Evdokimov Moscow State University of Medicine and Dentistry, Delegatskaya St., 20, p.1, Moscow 127473, Russia

**Keywords:** additive manufacturing, fused deposition modeling, ABS plastic, alumina, PLA plastic, mechanical properties, composite materials

## Abstract

In this paper, a mathematical model for the description of the failure probability of filament and fused deposition modeling (FDM)-printed products is considered. The model is based on the results of tensile tests of filament samples made of polyacrylonitrile butadiene styrene (ABS), polylactide (PLA), and composite PLA filled with alumina (Al_2_O_3_) as well after FDM-printed products of “spatula” type. The application of probabilistic methods of fracture analysis revealed that the main contribution to the reduction of fracture probability is made by the elastic and plastic stages of the fracture curve under static loading. Particle distribution analysis of Al_2_O_3_ combined with fracture probability analysis shows that particle size distributions on the order of 10^−5^ and 10^−6^ mm decrease the fracture probability of the sample, whereas uniform particle size distributions on the order of 10^−1^ and 10^−2^ mm do not change the distribution probability. The paper shows that uneven distribution of Al_2_O_3_ fillers in composite samples made using FDM printing technology leads to brittle fracture of the samples.

## 1. Introduction

In recent years, one of the main trends in industrial production has been the use of new technologies to create low-cost, high-quality products. In addition to achieving high product quality and low production costs, an important factor is the reduction of the time required to create the final product—“from idea to finished product”—to the minimum possible. These conditions bring additive manufacturing, which was developed as a technology for rapid prototyping, to the forefront.

In 2015, the International Organization for Standardization (ISO), together with the American Society for Testing and Materials (ASTM), issued a new international standard, ISO/ASTM 52900:2015, which sets out the terms used in additive manufacturing [1]. It also presents the classification of additive manufacturing technologies into groups according to the type of raw material, deposition technique, and method of melting or curing the material. The oldest and, consequently, most mature additive manufacturing technologies are Stereolithography (SLA, 1984), Selective Laser Sintering (SLS, 1987), and Fused Deposition Modeling (FDM, 1988) being the most common in the world [2,3,4,5]. For more than 30 years of FDM-printing technology existence, a great number of thermoplastics [2,3,4,5,6] designed for layer-by-layer printing of different items, from children’s toys to functional elements of aircraft structures, have been designed and developed.

Among the list of materials, polyacrylonitrile butadiene styrene (ABS) and polylactide (PLA) are the most common. The main distinguishing feature of ABS and PLA thermoplastics is their relatively low melting point. In the case of PLA plastic, the printing temperature is between 130–180 °C, whereas for ABS plastic, it is 230–260 °C. PLA plastic is an environmentally friendly material with low shrinkage during solidification and relatively low cost. ABS resists most solvents (except acetone) and moisture and has a relatively high thermal resistance between 90° and 110°. The main technological difference between ABS and PLA is the high shrinkage (about 0.8% by volume) of ABS plastic in the manufacture of products by FDM printing technology [7]. Along with classic materials, various composite materials containing ceramic, wood, metal, and carbon fillers are used, while the basis (matrix) filament (wire of different diameters, obtained by extrusion of thermoplastic pellets or powder) consists of classical thermoplastics including polylactide.

In the discussed work, PLA filled with alumina (Al_2_O_3_) particles is investigated. Polylactide is primarily attractive as a substrate due to its high stiffness and strength. In addition, PLA is a bio-based, biodegradable, and biocompatible polymer. The introduction of additional elements such as ceramic, carbon, wood, or metal into the filament is intended to increase its mechanical characteristics. However, improving the mechanical performance of the filament is associated with a number of technological difficulties. The most basic difficulty is obtaining a uniform distribution of these fillers along the entire length of the filament produced and throughout the entire volume of the samples printed from it.

When considering ABS and PLA plastics, the main factor affecting the mechanical properties of filament and final products is the presence of moisture, air bubbles, and the anisotropy of the plastic properties. Features of possible defects in the structure filament and products manufactured by FDM printing technology from ABS and PLA plastics allow the application of deterministic methods to predict the mechanical characteristics of the final products [8]. However, the random nature of defect distribution in ABS and PLA plastics and the distribution of Al_2_O_3_ particles in polylactide can lead to random behavior of strength and ductility of the material with a wide confidence interval along the entire length of the filament. The use of materials with non-uniform mechanical properties in the fabrication of FDM-printed products leads to an uneven distribution of mechanical properties throughout the product and, as a consequence, to the random nature of the fracture. With such a behavior of the initial material (filament) and the results of its use (final products), the best method to describe the process of material and product failure is a method based on probability theory [9].

The aim of this work is to find a mathematical model of the failure probability of filament and parts made by FDM printing technology from ABS, PLA, and prepared filament filled with alumina particles. The model is based on the results of static tensile tests filament and 3D printed samples. Based on previously published works, the content of ceramic was chosen around 60% [10,11,12,13,14,15].

## 2. Materials and Methods

Commercially available powders of Al_2_O_3_ (Plasmotherm Ltd., Moscow, Russia, with d_50_ = 40 µm) and PLA (eSun Ltd., Shenzhen, China, with an average particle diameter of 35 μm) were chosen as starting materials. For ceramic-polymer filament fabrication, the mixture of 60 vol. % of Al_2_O_3_ and 40 vol.% PLA was prepared in a PM 100 planetary mill (Retsch, Haan, Germany) for 2 h in distilled water. The resulting mixture was dried in a vacuum desiccator VO 400 (Memmert, Schwabach, Germany) for 24 h and then sieved on a vibratory sieve shaker AS 200 Basic (Retsch, Haan, Germany) through a sieve with a mesh size of 63 µm. The prepared mixture was pressed into pellets on a water-cooled hot mounting press OPAL 480 (ATM, Mammelzen, Germany). The obtained pellets were then loaded into the hopper of a Wellzoom tabletop extruder (Shanghai, China), and a filament was produced for 3D printing at 220 °C. Samples for mechanical studies were printed on a Black Widow 3D printer (Tevo 3D, Guangdong, China) at the following printing modes: nozzle temperature 200 °C, table heating temperature 70 °C, nozzle diameter 0.6 mm, layer height 0.4 mm, printing speed 10 mm/s, filling 100%. Tensile tests were carried out using a floor model universal electrodynamic testing machine Electropuls E10000 (Instron, Norwood, MA, USA) equipped with a 10 kN capacity load cell, and load weighing accuracy was ±0.5%. The movement speed of the machine grips was 2 mm/min, and the geometry of the 3D printed samples for tensile testing were fabricated with reference to the Type 1B specimens of the ISO 527-2:2019 standard [16]. The commercial PLA and ABS filaments (Bestfilament, Moscow, Russia) and printed samples from these plastics were also examined for comparison with produced filament. The materials used in this study are presented in Table 1.

**Figure 1:**
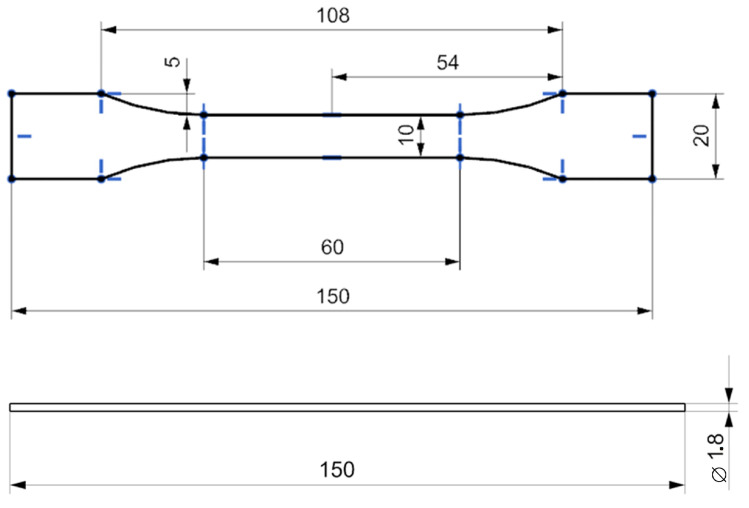
3D printed specimens (**top**) and filaments (**bottom**) for tensile testing.

The dimensions and geometry of the FDM printed specimens according to the ISO 527-2:2019 standard [16] and filaments for tensile testing are shown in Figure 1.

Six specimens were tested for each configuration to evaluate values of the tensile strength and Young’s modulus. All tests were conducted at room temperature 22 ± 1 °C and at 45 ± 5% relative humidity. According to the results of the type 1B specimens, 60Al_2_O_3_/40PLA and PLA rejected one result each, and ABS plastic rejected two results, respectively.

## 3. Results

**Figure 2:**
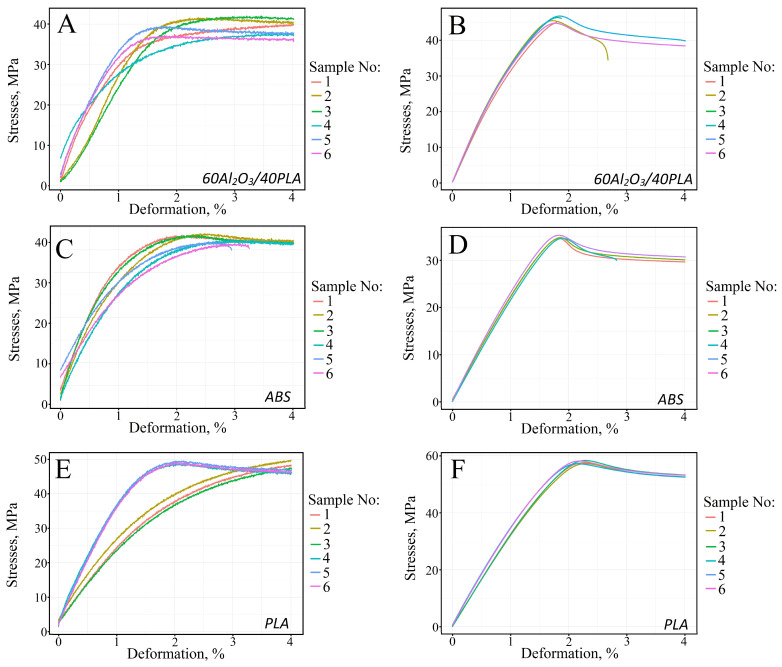
Tensile static curves for filaments (**A**,**C**,**E**) and printed (**B**,**D**,**F**) samples.

Figure 2A–F shows the static tensile test results of the filaments and samples printed from ABS, PLA, and polymer-ceramic filaments.

**Figure 3:**
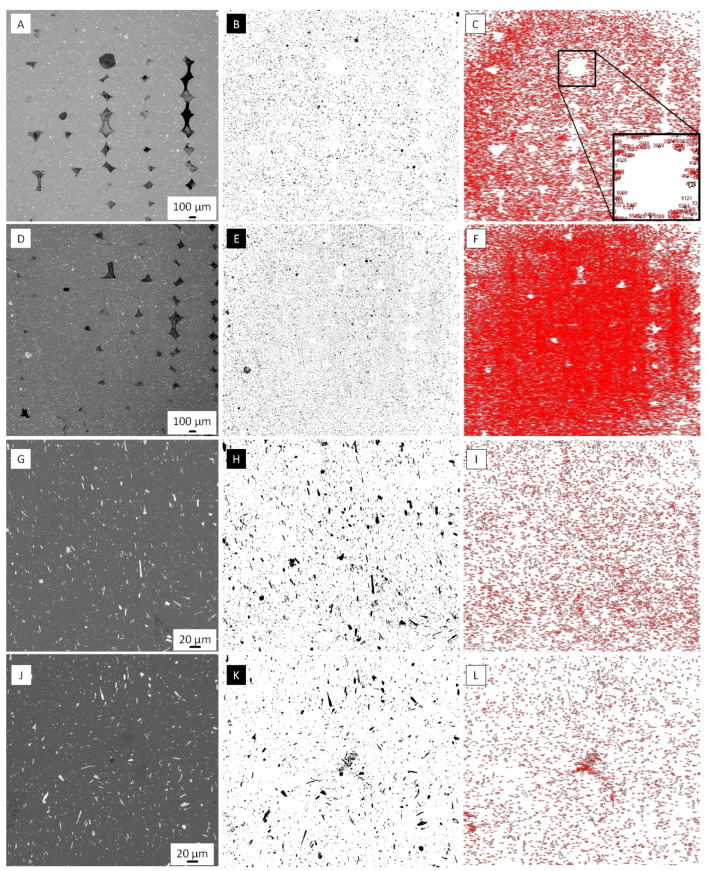
SEM images (**left column**), inverted images (**middle column**), and numbered ceramic fillers of 3D-printed 60Al_2_O_3_/40PLA samples numbered 1 (**A**–**F**) and 2 (**G**–**L**). The square area denotes where the representative close-up (**bottom right corner**) of numbered ceramic fillers was taken (**C**).

The analysis of static stretch diagrams of ABS, PLA, and 60Al_2_O_3_/40PLA samples shows that the complete failure of the specimens occurs at a strain of 4% (Figure 2C–E). The same fracture strain value is obtained for the printed specimens (Figure 2B, D, and F). The exceptions are samples 1–3 (Figure 2B), samples 5 and 6 (Figure 2C), and sample 3 (Figure 2D). For samples 1 and 3, produced from ceramic-polymer filament using FDM printing technology, the value of deformation at break is 1.8%, for samples 2–2.7% (Figure 2B). Samples 5 and 6 (Figure 2C) fracture at 2.9% and 3.5% strain, respectively, and sample 3 (Figure 2D) at 2.8% strain. An elastic-plastic strain diagram is observed for the three PLA thermoplastic filament samples (Figure 2E, samples 1–3). In the case of specimens 1 and 3 (Figure 2B), the strain curve describes the fracture process of a specimen made of a non-plastic material and contains only the elastic region of the diagram. Figure 3 shows a graphical analysis of the fracture region of a 3D-printed 60Al_2_O_3_/40PLA specimen (Figure 2B). In order to confirm the percentage of aluminum oxide in the polymer, the colors of the SEM images of the fracture surfaces (Figure 3 left column) were inverted to the opposite (Figure 3 middle column); the white ceramic is marked with black color, and the rest is marked with white color. Then each dark point was numbered in red (Figure 3, right column). It should be noted that for better observation of ceramic fillers, the fracture surfaces were polished.

The graphical analysis of the fracture region of printed 60Al_2_O_3_/40PLA sample number 1 shows a high concentration of ceramic fillers in the fracture zone and heterogeneities during FDM printing, which together lead to brittle fracture of the sample. Similar behavior was observed in the graphical analysis of the fracture area of printed 60Al_2_O_3_/40PLA sample number 2 (Figure 3G–L). Table 2 shows the mean values of tensile strength and offset yield strength derived from the analysis of strain curves of filament and printed samples (Figure 1).

A comparison of the tensile and offset yield strengths with previously obtained results [4,17,18,19] shows that the values presented in Table 2 (columns 3 and 4) are close to the previously obtained values [4,17], except for the behavior of conditional plasticity limit. The reasons for the differences in σ_H_ and σ_0.2_ values may be due to the presence of dyes in the filament, heterogeneity in the structure of the filament itself, and, when examining spade-type samples, the technological modes of FDM printing. The latter statement is confirmed by the presented results, especially by the increase in yield and tensile strength of the printed samples (with the exception of the average value of tensile strength for printed ABS samples). The greatest increase in strength properties is observed in printed 60Al_2_O_3_/40PLA samples. Generalization of the tensile test results shows that ABS plastic has uneven mechanical properties, randomly distributed along the length of filament, which affects the properties of printed samples. In the case when the ceramic-polymer filament is used for FDM may be an uneven distribution of ceramic fillers over the whole volume of the sample and, as a consequence, the presence of areas prone to brittle fracture. The results obtained from static tensile testing of the filament and printed specimens confirm the random nature of the distribution of mechanical properties from the filament to the final product. The exception in the given results is the filament and products obtained by FDM printing technology from ABS plastic. Studies of fracture processes of composite materials and materials with non-popular properties appear to be incomplete without mathematical modeling. Previously, several attempts have been made to simulate the fracture processes of type 1B and filament specimens on the basis of mixture theory and the molten deposit method [4,8]. In the considered work, an alternative method of modeling aimed at establishing the risks (probability) of fracture is used. It is more often used in the analysis of the stability of complex structures. An independently developed software written in the R language was used to build the model.

In [9], the results of mathematical modeling of fracture probability of pre-cyclically loaded specimens are presented, and it is shown that the description of the stress distribution function is more accurate in the interval description of the fracture diagram. Four main intervals of the fracture diagram of the specimen under static loading are distinguished:

1. from 0 to the conditional limit of proportionality (*σ_lp_*);
2. from the notional limit of proportionality (*σ_lp_*) to the yield strength(*σ_ys_*);
3. from yield strength (*σ_ys_*) to tensile strength (*σ_H_*);
4. from tensile strength (*σ_H_*) to fracture strength (*σ_fs_*).

**Figure 4:**
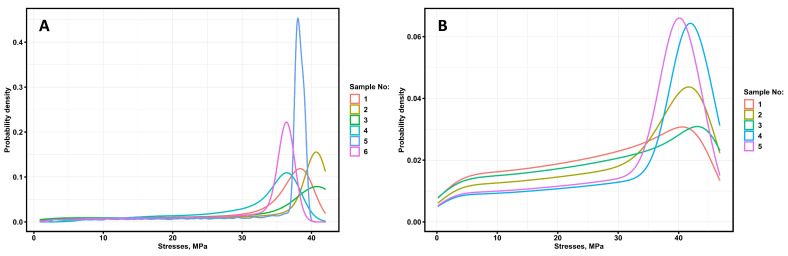
Fracture probability density functions of the 60Al_2_O_3_/40PLA filaments (**A**) and printed samples (**B**).

Figure 4 shows the behavior of the stress probability density when testing filament specimens and printed 60Al_2_O_3_/40PLA samples, corresponding to the diagrams in Figure 1A,B.

For practical applications, the failure probability of printed samples is of the most interest. Table 3 presents the results of the distribution type analysis at the four sites. The analysis was performed based on a comparison of theoretical and actual distributions of fracture probability density function, with criteria of the closeness of two distributions being Akaike and Bayes criterion.

The static fracture diagrams of printed 60Al_2_O_3_/40PLA specimens 1 and 3 have no plastic region and are subject to brittle fracture. For specimens 4 and 5, a normal distribution is observed in the areas from the limit of proportionality to the yield strength, and for specimen 4, the closest statistical distribution is the Cauchy distribution of stresses. For the first and second specimens, the probability density functions for a fracture will be of the form:

(1) $$f\left(\sigma \right)=\{\begin{array}{c} \left(\frac{a}{b}\right)*{\left(\frac{\sigma }{b}\right)}^{\left(a-1\right)}*exp(-{\left(\frac{\sigma }{b}\right)}^{a})\;for\;0\leq \sigma <\sigma_{lp}\\ \left(\frac{a}{b}\right)*{\left(\frac{\sigma }{b}\right)}^{\left(a-1\right)}*exp(-{\left(\frac{\sigma }{b}\right)}^{a})\;for\;\sigma_{lp}\leq \sigma <\sigma_{ys}\\ \left(\frac{a}{b}\right)*{\left(\frac{\sigma }{b}\right)}^{\left(a-1\right)}*exp(-{\left(\frac{\sigma }{b}\right)}^{a})\;for\;\sigma_{ys}\leq \sigma \leq \sigma_{H} \end{array}$$

where *a*—distribution shape parameter; *b*—scale factor; *σ*—stresses, MPa.

Table 4 shows the numerical values of the distribution shape parameter and scale factor for printed 60Al_2_O_3_/40PLA samples 1 and 2.

Given the uniformity and continuity of the specimen fracture probability density function in a static test, the total probability density function will be the sum of the probability densities, and the failure probability of the specimen will be the sum of the probabilities at each site. Non-normalized equations for stresses varying from 0 to the conditional fracture limit:

(2) $$\begin{array}{c} P\left(\sigma \right)=3-exp\left(-0.21483*\sigma_{1}^{2.508593}\right)-0.9\left(9\right)\\ \;\;\;\;\;\;*exp\left(-0.00063*\sigma_{2}^{2.370878}\right)-0.9\left(9\right)\\ \;\;\;\;\;\;*exp\left(-7.767*10^{-25}*\sigma_{3}^{14.86595}\right) \end{array}$$

(3) $$\begin{array}{c} P\left(\sigma \right)=3-exp\left(-0.213775*\sigma_{1}^{2.50677}\right)-0.9\left(9\right)\\ \;\;\;\;\;\;*exp\left(-0.001100*\sigma_{2}^{2.926659}\right)-0.9\left(9\right)\\ \;\;\;\;\;\;*exp\left(-8.28*10^{-9}*\sigma_{3}^{6.37499}\right)-exp(-2.88\\ \;\;\;\;\;\;*10^{-28}*\sigma_{4}^{17.73303}) \end{array}$$

where 0 ≤ *σ_1_* ≤ *σ_lp_*; *σ_lp_* ≤ *σ_2_* ≤ *σ_ys_*; *σ_ys_* ≤ *σ_3_* ≤ *σ_H_*; *σ_H_* ≤ *σ_4_* ≤ *σ_fs_*.

**Figure 5:**
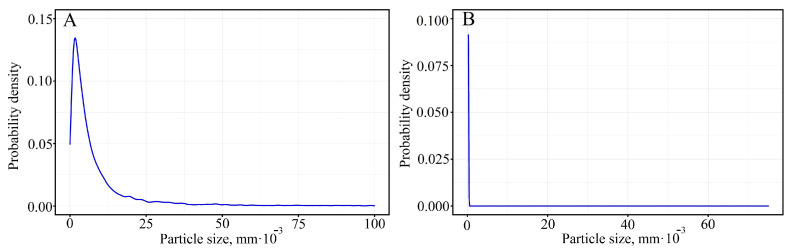
Probability density function of alumina particle size distribution in 3D printed 60Al_2_O_3_/40PLA sample 1 (**A**) and 2 (**B**).

Equations (2) and (3) describe the failure probability of specimens 1 and 2, respectively. The analysis of the equations shows that the major contribution to the failure probability is made by the second term of the equations obtained in the area from 0 to the conventional limit of proportionality, the third major contribution to the failure probability is made in the area from the conventional limit of proportionality to the conventional yield strength. The calculation of fracture probability according to Equation (3) at zero stresses gives an infinitesimal value of fracture probability and at values close to fracture stress, 72.3%, while the fracture probability of the first sample is 21.6%. An empirical particle distribution function was obtained for printed ceramic-polymer No.1 and No.2 specimens (Figure 5) to account for the presence of alumina fillers in the PLA.

Evaluation of the closest theoretical statistical distribution using the Akaike and Bayes criteria shows that for sample 1, the closest distribution is the Cauchy probability density function, and for sample 2, the Lognormal distribution, which has the form:

(4) $$f\left(x\right)=\frac{1}{x*sd\sqrt{2\pi }}exp\left(-\frac{1}{2}*{\left(\frac{\ln\left(10*x\right)-\mu }{sd}\right)}^{2}\right)$$

(5) $$f\left(x\right)=\frac{1}{\pi *s*\left(1+{\left(\frac{10^{3}*x-l}{s}\right)}^{2}\right)}$$

where *l* is the shift factor; *s* is the scale factor; *sd* is the variance of the lognormal distribution; *μ* is the shift factor of the lognormal distribution; *x* is Al_2_O_3_, the particle size in mm. For the distribution of particles in the fracture region of sample 1, the lognormal shift factor is 1.58, and the variance is 1.40. For the distribution of particles in the fracture region of sample 2, the shear coefficient is 5.51 · 10^−3^, and the scale factor is 3.44 · 10^−8^. The probability function for Lognormal distribution and Cauchy distribution of fracture area of specimens 1 and 2 will have the form:

(6) $$P\left(x\right)=0.5*erf\left(\sqrt{2}*\left(0.358967*\ln\left(10*x\right)-0.563666\right)\right)$$

(7) $$P\left(x\right)=0.00031831*arctg\left(2.904895*10^{10}*x-160059.724645\right)$$

where *x* is the particle size of Al_2_O_3_ in mm.

Considering the fact that the Al_2_O_3_ particle distribution and the fracture diagram belong to the same sample and correlate with each other, the total fracture probability will be of the form:

(8) P(σ,x)=P(σ)∗P(x)

Analysis of the resulting equation shows that the probability of failure does not exceed 0.1% at all stress values (up to the specimen fracture stress) and at all filler particle size values in specimen 1. In specimen 2, the failure probability is 0.096%, with a more complex structure of the failure probability function. The analysis of the probability analysis results shows that the minimum fracture probability of sample 1 is lower than that of sample 2; sample 2 has a more uniform particle size distribution than sample 1.

## 4. Conclusions

The results of static tensile tests showed that ABS plastic filament has uneven mechanical properties distributed randomly along the length of the filament, which affects the properties of the product obtained by FDM printing. When using for FDM printing fabricated ceramic-polymer filament, the non-uniform distribution of ceramic fillers over the whole volume of the sample can be observed, and as a result, the presence of areas prone to brittle fracture. Inheritance of the results and the greatest uniformity of mechanical properties are shown by the filament made of PLA plastic. The static fracture diagrams of the PLA filament specimens are divided into two groups (three specimens in each group), whereas the printed PLA specimens have diagrams with close stress-strain relations. Thus, the mechanical properties of PLA plastic are most uniform, compared to ABS plastic, along the length of the filament and when making products using FDM printing technology. Mathematical modeling using probabilistic analysis methods shows that the main contribution to reducing the fracture probability of PLA specimens filled with Al_2_O_3_ particles is due to the elastic and plastic components of the equation describing the fracture probability. The comparison of the contributions decreasing the fracture probability of printed 60Al_2_O_3_/40PLA specimens shows that the contribution of the elastic-plastic region of specimen 2 is higher than that of specimen 1. The reasons for such behavior of the elastic-plastic contributions in the static fracture diagram for specimens fabricated of PLA filled with Al_2_O_3_ are subject to further investigation. Combined analysis of fracture probability and Al_2_O_3_ particle size distribution shows that small particles (order 10^−5^ and 10^−6^ mm) of almost equal size present in the second sample (indicated by Cauchy distribution) reduce the fracture probability of the sample to an almost constant value, whereas more homogeneous (lognormal distribution) and larger particles (order 0.1 mm) maintain a complex distribution probability curve and raise the minimum probability value. Further research will focus on expanding the factors influencing the probability of failure, and an attempt will be made to classify the main thermoplastics used in FDM printing technology based on the probability of failure. 

## Figures and Tables

**Table 1:** Full names and abbreviations of all studied specimens.

Materials	Abbreviation
Ceramic-polylactide	60Al_2_O_3_/40PLA
Acrylonitrile butadiene styrene	ABS
Polylactide	PLA

**Table 2:** Mean tensile and offset yield strengths.

Material Type	Geometry	Mean Tensile Strength, σ_H_ [MPa]	Mean Offset Yield Strength, σ_0.2_ [MPa]	Mean Tensile Strength, σ_H_ [MPa]	Mean Offset Yield Strength, σ_0.2_ [MPa]
Ceramic-polylactide	Filaments	40.20 ± 2.18	32.90 ± 5.66	-	-
ABS		37.60 ± 1.12	28.00 ± 3.09	35–110 [17]	25–50 [17]
PLA		49.80 ± 1.03	32.50 ± 6.10	52.30 [18]	12.73 [18]
Ceramic-polylactide	Type 1B [16]	45.70 ± 1.09	35.20 ± 10.60	-	-
ABS		34.70 ± 0.48	32.50 ± 6.82	34.69 [19]	29.48 [19]
PLA		57.70 ± 0.81	49.60 ± 19.22	44.67 [17]	10.51 [17]

**Table 3:** Stress distribution types in static tensile stress sections of 3D-printed 60Al_2_O_3_/40PLA specimens.

Sample Number	From 0 to σ_lp_	From σ_lp_ to σ_ys_	From σ_ys_ to σ_H_	From σ_H_ to σ_fs_
1	Weibull	Weibull	Weibull	
2	Weibull	Weibull	Weibull	Weibull
3	Weibull	Weibull	Weibull	
4	Weibull	Normal	Cauchi	Weibull
5	Weibull	Normal	Weibull	Weibull

**Table 4:** Values of the shape parameter and scale factor for printed 60Al_2_O_3_/40PLA samples 1 and 2 distributions.

Coefficient Value at an Interval	Sample No. 1	Sample No. 2
*a* on an interval from 0 to *σ_lp_*	2.51	2.51
*b* on an interval from 0 to *σ_lp_*	1.85	1.85
*a* on an interval from *σ_lp_* to *σ_y_*_s_	2.37	2.93
*b* on an interval from *σ_lp_* to *σ_ys_*	22.39	10.25
*a* on an interval from *σ_ys_* to *σ_H_*	14.87	5.09
*b* on an interval from *σ_ys_* to *σ_H_*	41.86	38.43
*a* on an interval from *σ_H_* to *σ_fs_*	-----------	17.73
*b* on an interval from *σ_H_* to *σ_fs_*	-----------	35.73

## Data Availability

Not applicable.
